# Angioarchitectural alterations in the retina and choroid in frontotemporal dementia

**DOI:** 10.1371/journal.pone.0312118

**Published:** 2024-11-08

**Authors:** Ariana Allen, Cason B. Robbins, Suzanna Joseph, Angela Hemesat, Anita Kundu, Justin P. Ma, Alice Haystead, Lauren Winslow, Rupesh Agrawal, Kim G. Johnson, Andrea C. Bozoki, Sandra S. Stinnett, Dilraj S. Grewal, Sharon Fekrat

**Affiliations:** 1 iMIND Study Group, Duke University School of Medicine, Durham, NC, United States of America; 2 Department of Ophthalmology, Duke University School of Medicine, Durham, NC, United States of America; 3 National Healthcare Group Eye Institute, Tan TOCK SENG Hospital, Singapore, Singapore; 4 LKC School of Medicine, Nanyang Technological University, Singapore, Singapore; 5 Department of Neurology, Duke University School of Medicine, Durham, NC, United States of America; 6 Department of Neurology, University of North Carolina School of Medicine, Chapel Hill, NC, United States of America; IRCCS San Raffaele Scientific Research Institute, ITALY

## Abstract

**Objective:**

Frontotemporal dementia (FTD) is a progressive neurodegenerative disorder that affects the frontal and temporal lobes of the brain, leading to cognitive decline and personality changes. The objective of this cross-sectional study was to characterize angioarchitectural changes in the retina and choroid of individuals with FTD compared to cognitively normal controls using optical coherence tomography (OCT) and OCT angiography (OCTA).

**Methods:**

Cross-sectional comparison of patients with FTD and controls with normal cognition. All participants underwent Mini-Mental State Examination (MMSE) at the time of imaging. Outcome measures included OCT parameters: retinal nerve fiber layer (RNFL) thickness, ganglion cell layer-inner plexiform layer (GC-IPL) thickness, central subfield thickness (CST), subfoveal choroidal thickness (SFCT), choroidal vascularity index (CVI); and OCTA superficial capillary plexus parameters: foveal avascular zone (FAZ) area, 3x3mm and 6x6mm macular perfusion density (PD) and vessel density (VD), 4.5x4.5mm peripapillary capillary perfusion density (CPD) and capillary flux index (CFI). Generalized estimating equation analysis was used to account for the inclusion of 2 eyes from the same participant.

**Results:**

29 eyes of 19 patients with FTD and 85 eyes of 48 controls were analyzed. In FTD, 3x3mm macular PD (p = 0.02) and VD (p = 0.02) and CFI (p = 0.01) were reduced compared to controls. There was no difference in average 4.5x4.5mm CPD, RNFL thickness, GC-IPL thickness, CST, SFCT, CVI, FAZ, or 6x6mm VD or PD between FTD and controls (all p > 0.05); however, there was a trend toward lower macular 6x6mm PD and VD in patients with FTD.

**Conclusion:**

Decline of peripapillary and macular OCT and OCTA parameters merit further investigation as potential biomarkers for FTD detection. Noninvasive retinal and choroidal imaging may hold promise for earlier detection, and future longitudinal studies will clarify their role in monitoring of FTD.

## Introduction

Declining cognition with age is becoming an increasingly significant limitation to quality of life and safety. The number of people living with dementia has more than doubled since 1990, and in 2016, dementia was the fifth leading cause of death globally [1]. Of non-Alzheimer’s disease (AD) dementias, frontotemporal dementia (FTD) is a common cause of early-onset (age < 65 years) dementia [2]. FTD is a clinical syndrome with varying presentations categorized into predominantly behavioral (disinhibition, perseveration, inappropriate behaviors, marked personality changes) or language (aphasia) disturbances [3]. Due to its clinical heterogeneity and overlap with other dementias, FTD is often misdiagnosed or recognized late, limiting the potential for research into FTD therapies [2–5].

Due to associated neuropsychiatric and behavioral symptoms in patients with FTD, there is a higher psychiatric morbidity and caregiver burden compared to other dementias [2, 3]. Prompt diagnosis and monitoring has the potential to improve the quality of life for both patients and caregivers. FTD-associated neuropathology can be definitively diagnosed post-mortem; however, due to the heterogeneity of FTD syndromes, the correlation between clinical presentation and neuropathology is variable [4–6]. Available FTD biomarkers include neuroimaging biomarkers (MRI, FDG-PET), fluid biomarkers (cerebrospinal fluid [CSF] analysis), and genetic biomarkers consisting of chromosome 9 open reading frame 72 (c9ORF72), microtubule-associated protein tau (MAPT), and progranulin (GRN) genes [4]. These existing FTD markers are limited by insensitivity, lack of specificity, lack of consensus regarding disease staging, progression, and severity, and by expense [4]. As such, more sensitive, non-invasive, and cost-effective biomarkers to detect and monitor FTD are greatly needed.

A growing body of literature has demonstrated a correlation between microvascular and structural changes in the retina and neurodegenerative changes in the brain. Optical coherence tomography (OCT) and OCT angiography (OCTA) have been explored as potential noninvasive biomarkers in neurodegenerative diseases; however, data is lacking on retinal and choroidal microvascular and structural changes in FTD in comparison to controls [7, 8].

In this study, we use OCT and OCTA to characterize differences in the retinal and choroidal angioarchitecture in eyes of individuals with FTD compared to eyes of controls with normal cognition.

## Methods

This cross-sectional study was approved by the Duke Health Institutional Review Board (IRB) (Pro111831). Written informed consent was obtained from all eligible individuals or their legally authorized representative before enrollment. Capacity to provide consent was assessed by the referring neurologist. If the participant was deemed unable to provide informed consent, a legally authorized representative reviewed the informed consent and signed on their behalf. This consent procedure was approved by the Duke IRB. This study abided by all tenets set forth in the Declaration of Helsinki regarding human subjects and complied with the Health Insurance Portability and Accountability Act of 1996.

### Study participants

Individuals with a clinical diagnosis of FTD were recruited from the Duke Neurology Disorders Clinic in Durham, NC by the iMIND Research Group team. Clinical diagnosis of FTD was confirmed in association with accepted clinical guidelines (A.C.B., K.G.J.) prior to enrollment. The guidelines specify behavioral or language changes, MRI changes with areas of temporal or frontal atrophy, and a decrease in functioning [9–11]. Behavioral changes include three or more out of six clinically discriminating features: disinhibition, apathy/inertia, loss of sympathy/empathy, perseverative/compulsive behaviors, hyperorality and dysexecutive neuropsychological profile [10]. Language changes include nonfluent/agrammatic, semantic, and logopenic changes [11]. Control participants without cognitive dysfunction and a decrease in functioning were volunteers who were enrolled from the Duke Neurology Disorders Clinic or the Duke Alzheimer’s Disease Prevention Registry of community-dwelling volunteers with normal cognition based on an extensive neuropsychologic testing battery.

Exclusion criteria included a history of non-FTD-associated dementias, diabetes, uncontrolled hypertension, glaucoma, retinal or optic nerve pathology, and corrected Snellen visual acuity worse than 20/40 at the time of image acquisition and spherical equivalent (SEQ) of less than -6 diopters (D) or greater than +6D. Participants also underwent ultra-widefield (UWF) scanning laser ophthalmoscopy (Optos California, Optos, Marlborough, MA) to further screen for vitreoretinal pathology. Participants found to meet any exclusion criterion by reported history, medical records, or UWF or OCT imaging were excluded.

All study participants underwent cognitive evaluation using the Mini-Mental State Examination (MMSE; score range 0–30, with higher scores indicating better performance) at the time of image acquisition. Years of education were determined per patient and caregiver report starting from first grade onwards.

### OCT and OCTA image acquisition

Each participant underwent nonmydriatic imaging with the Zeiss Cirrus HD-5000 Spectral-Domain OCT with AngioPlex OCTA, version 11.0.0.29946 (Carl Zeiss Meditec), which uses an optical microangiography algorithm for analysis and eye tracking to reduce motion artifact [12].

For each eye, a 512 × 128-μm macular cube, a 200 × 200-μm optic disc cube, and a high-definition, 21-line enhanced depth imaging (EDI) foveal scan was acquired. Images with low signal strength (less than 7/10 for macular cube and optic disc cube, less than 6/10 for 21-line EDI), motion artifact, segmentation artifact, or focal signal loss were excluded. Mean retinal nerve fiber layer (RNFL) thickness (in μm) was measured over a 3.46-mm diameter circle centered on the optic disc. Mean ganglion cell layer-inner plexiform layer (GC-IPL) thickness (in μm) was quantified over the 14.13-mm^2^ elliptical annulus area centered on the fovea. Central subfield thickness (CST [in μm]) was quantified as the thickness between the inner limiting membrane and retinal pigment epithelium at the fovea.

The subfoveal choroidal thickness (SFCT [in μm]) was assessed manually as a linear measurement from the hyperreflective line of the outer border of the retinal pigment epithelium perpendicularly to the hyperreflective sclerochoroidal junction on the EDI foveal scan by two trained masked iMIND graders with any discordant measures adjudicated by a third experienced grader (D.S.G.). The choroidal vascularity index (CVI) was calculated by applying image binarization techniques to extended-depth imaging foveal scan that were analyzed using the Comprehensive Ocular Imaging Network (COIN) portal (www.ocularimaging.net) [13].

OCTA parameters of the superficial capillary plexus (SCP) were assessed using 3x3-mm and 6x6-mm OCTA images centered on the fovea, as well as 4.5x4.5-mm images centered on the optic nerve. Images were manually assessed by trained study staff, and images with poor scan quality (less than 7/10 signal strength index), motion artifact, segmentation artifact, or focal signal loss were excluded.

A thresholding algorithm was applied to OCTA *en face* images to create a binary skeletonized slab. Full-thickness retinal scans were segmented and the superficial capillary plexus, defined as the vasculature between the inner boundary of the internal limiting membrane and the outer boundary of the inner plexiform layer, was identified. The software detected the internal limiting membrane and calculated the inner plexiform layer as 70% of the distance from the internal limiting membrane to the estimated boundary of the outer plexiform layer, which was estimated to be 110 μm above the retinal pigment epithelium boundary [14].

For 3x3-mm scans, the foveal avascular zone (FAZ) area was determined; FAZ boundaries were automatically calculated by the Zeiss AngioPlex software and then manually reviewed to correct inaccurate boundaries or exclude those that could not be corrected. The vessel density (VD) and perfusion density (PD) were measured using an Early Treatment Diabetic Retinopathy Study (ETDRS) grid overlay. Vessel density was defined as the total length of perfused vasculature per unit area in the region of measurement. Perfusion density was defined as a percentage of area of perfused vasculature per unit area in a region of measurement. Both VD and PD were measured over the 3x3-mm ETDRS circle and ring, as well as the 6x6-mmm ETDRS circle and inner and outer rings.

For 4.5x4.5-mm scans, the radial peripapillary capillary (RPC) plexus, which runs parallel to the ganglion cells in the RNFL, was assessed by generating a binary vessel slab from the internal limiting membrane to the outer boundary of the RNFL. The capillary perfusion density (CPD), representing the percentage of perfused capillary in each defined area of interest, and capillary flux index (CFI), a unitless ratio representing the proportion of red blood cells in each vessel at a given point in time, were automatically calculated by the Zeiss software. Numerical data were collected from the imaging device software for statistical analysis. Images were reviewed by trained study staff for artifacts including motion, segmentation, signal loss, or decentration of the optic nerve head and poor-quality images were excluded prior to analysis.

### Statistical analysis

All statistical analyses were completed using SAS, version 9.4 (SAS Institute Inc).

Groups were compared using multivariable generalized estimating equations (GEE) by a statistician (S.S.S.), adjusting for age and sex as covariates. The GEE model was utilized to account for inclusion of 2 eyes from the same participant. Patient demographic variables were compared at the participant level across groups using the Chi-square test for categorical variables and the t-test or Wilcoxon rank sum test for continuous variables. An alpha of 0.05 was used to determine statistical significance. Conclusions were based on observed differences in population means.

## Results

A total of 44 eyes of 24 participants with FTD and 88 eyes of 48 controls with normal cognition were imaged. 15 FTD eyes and 3 control eyes were excluded due to poor image quality. We analyzed 32 eyes of 19 participants with FTD (63.2% men; mean [SD] age, 67.8 [12.3] years) and 85 eyes of 48 controls (62.5% men; mean [SD] age, 68.9 [10.9] years).

Table 1 describes the demographic and clinical characteristics of all participants. Individuals with FTD were similar to their control counterparts with regard to mean (SD) age, sex, and family history of dementia (47% vs 41%; p = 0.65). Compared to controls, years of education (15.6 [2.3] years vs 17.0 [2.1] years; p = 0.03) in FTD participants were lower, and MMSE scores of FTD participants were significantly lower (23.4 [6.7] vs 29.5 [1.2]; p<0.001).

**Table 1:** Demographics and clinical characteristics.

Variable	FTD	Control	P-value*
	N = 19	N = 48	
**Age** (mean ± SD)	67.79 ± 12.30	68.98 ± 10.92	0.700
**MMSE** (mean ± SD)	23.89 ± 6.35	29.46 ± 1.17	**<0.001**
**Education** (mean ± SD)	15.63 ± 2.31	17.00 ± 2.06	**0.030**
**Male sex** (N, %)	11 (63.2%)	30 (62.5%)	0.960
**Family history** (N, %)	8 (47.4%)	19 (41.3%)	0.653

Abbreviations: MMSE, mini-mental status exam

*P-value for age based on t-test. P-value for MMSE and education based on Wilcoxon rank sum test. P-value for categorical variables based on Chi-square test

**Fig 1:**
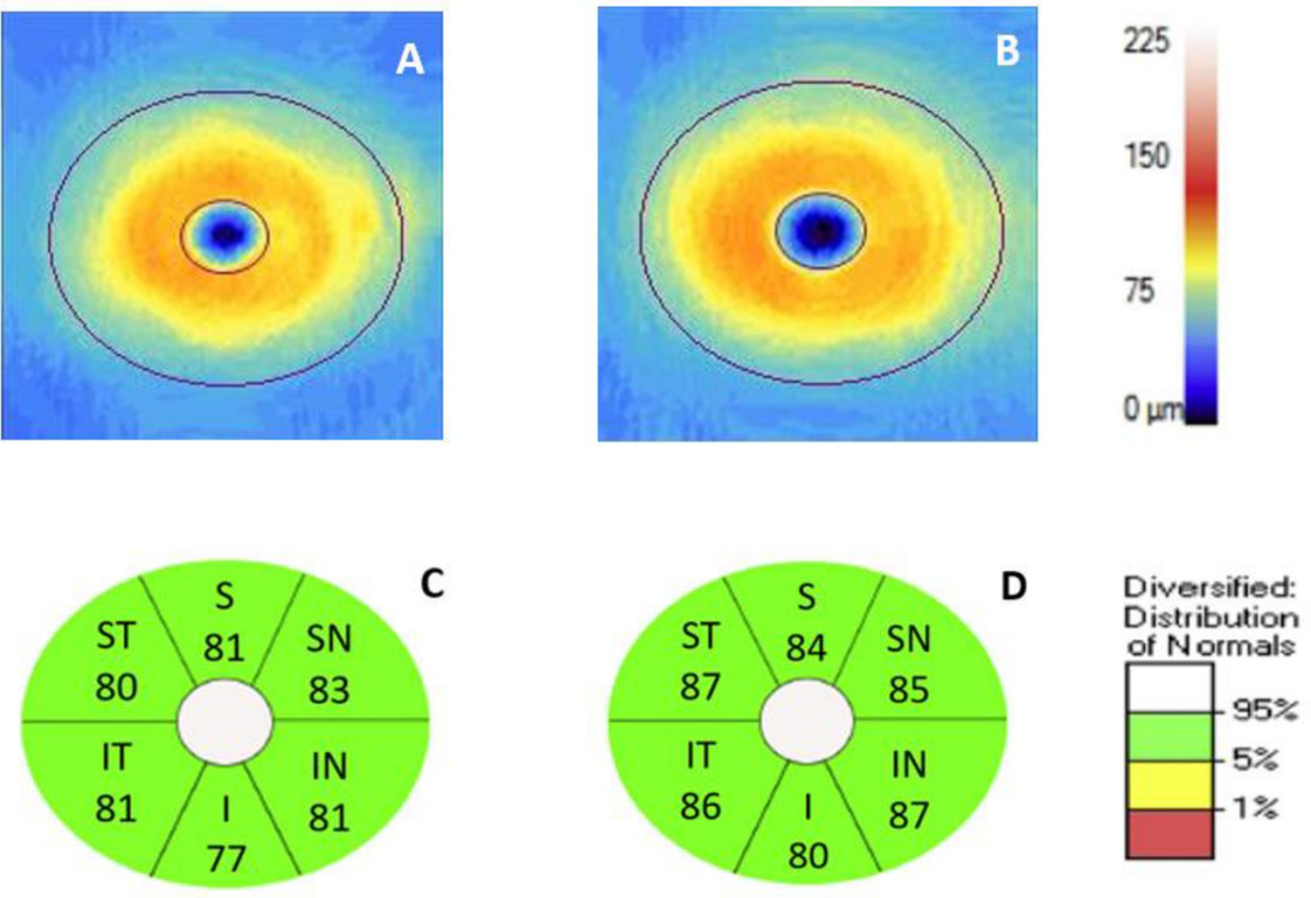
Representative ganglion cell analysis of the macula. Ganglion cell analysis (Carl Zeiss Meditec, Dublin, CA) of the macula of the left eye in an individual with frontotemporal dementia (FTD) (A) and the left eye of a control with normal cognition (B) with corresponding ganglion cell-inner plexiform layer thickness for the elliptical annular region of the FTD participant (C) and the control participant (D). I = inferior; IN = inferonasal; IT = inferotemporal; S = superior; SN = superonasal; ST = superotemporal.

Results of GEE analysis of the association of OCT and choroidal structural parameters with FTD diagnosis are shown in Table 2. Individuals with FTD did not differ significantly from controls in measures of OCT imaging, including RNFL thickness, GC-IPL thickness (Fig 1), CST, SFCT, and CVI (all p>0.05).

**Table 2:** Results of structural OCT parameter analysis in FTD participants and controls with generalized estimating equation analysis, adjusted for age and sex as covariates.

OCT Parameter Mean ± SD (N)	FTD	Control	P-value*
**CST**	265.2 ± 28.52 (N = 29)	265.0 ± 18.63 (N = 84)	0.988
**GC-IPL thickness**	75.37 ± 8.39 (N = 27)	77.55 ± 5.97 (N = 83)	0.324
**RNFL thickness**	89.45 ± 9.95 (N = 20)	88.15 ± 8.70 (N = 85)	0.907
**SFCT**	291.8 ± 61.51 (N = 29)	254.3 ± 71.88 (N = 85)	0.095
**CVI**	0.661 ± 0.014 (N = 28)	0.658 ± 0.017 (N = 85)	0.312

Abbreviations: SFCT, subfoveal choroidal thickness; CST, central subfield thickness; GC-IPL, ganglion cell-inner plexiform layer; RNFL, retinal nerve fiber layer; CVI, choroidal vascularity index

*P-value based on test of difference between means using generalized estimating equations (GEE) to account for correlation between eyes of the same patient, adjusted for age and sex.

**Fig 2:**
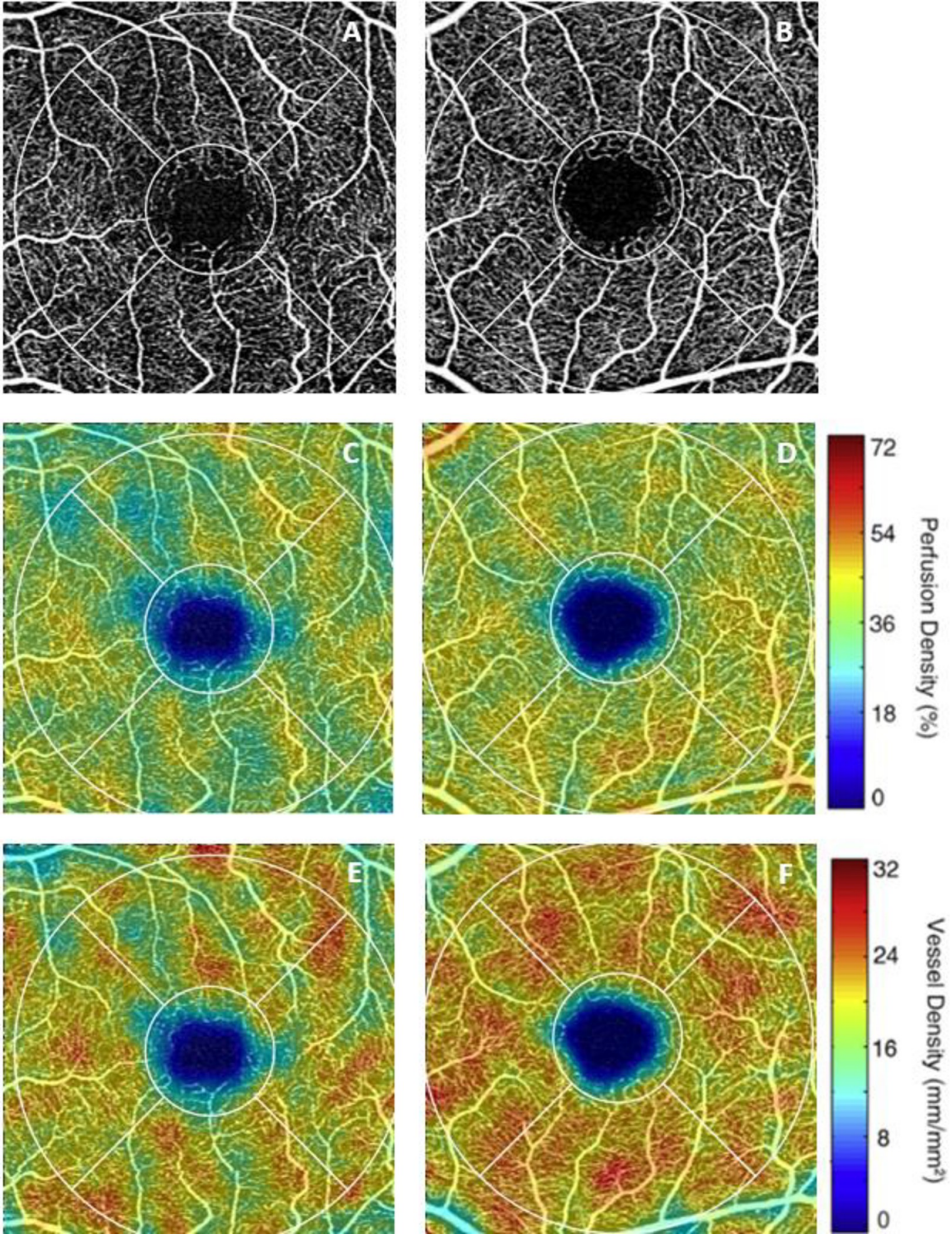
Representative 3x3-mm macular OCT angiography. 3x3-mm macular OCT angiography (OCTA) images of the superficial capillary plexus (SCP) of the left eye of an individual with frontotemporal dementia (FTD) (A) and the left eye of a control with normal cognition (B). Corresponding quantitative color maps of (C-D) perfusion density and (E-F) vessel density of the SCP, with the scale on the right. *Zeiss Cirrus HD-5000 Spectral-Domain OCT with AngioPlex OCTA software*, *version 11*.*0*.*0*.*29946 (Carl Zeiss Meditec)*.

**Fig 3:**
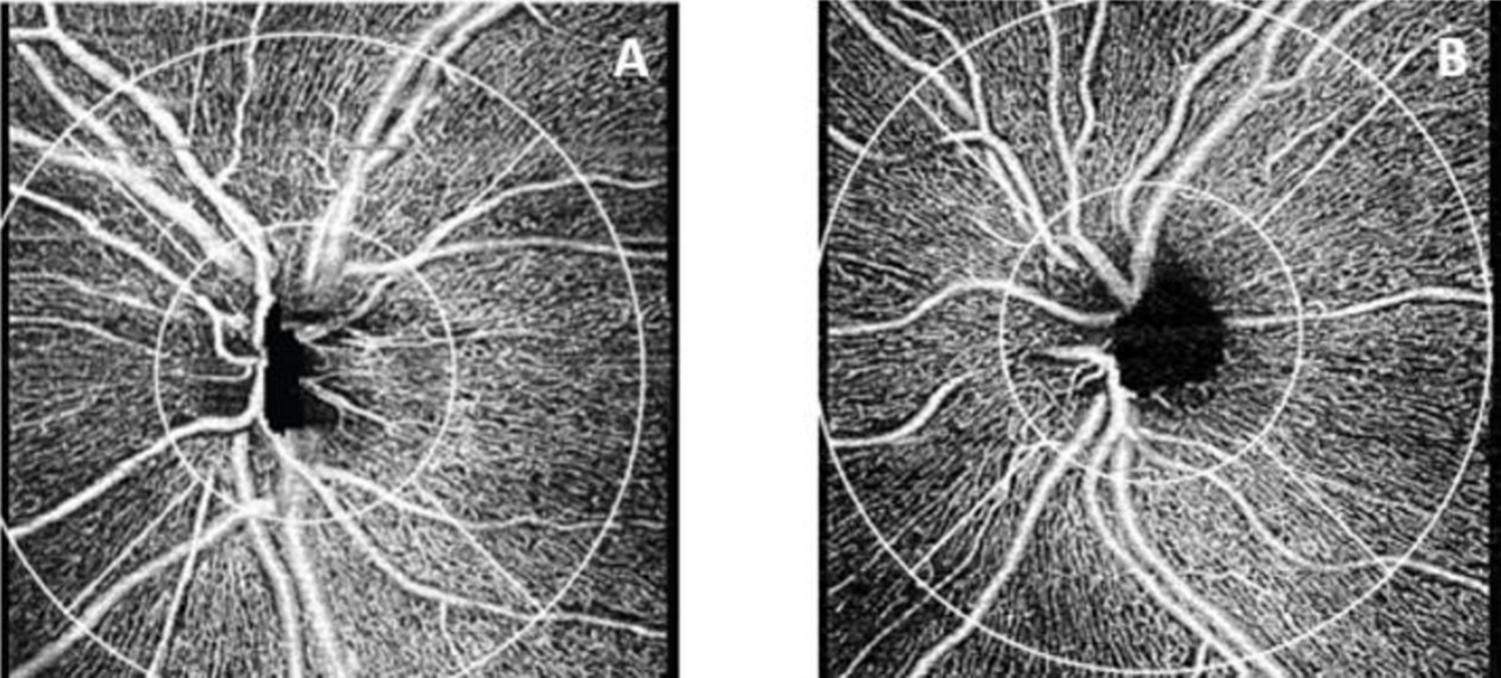
Representative 4.5x4.5-mm peripapillary OCT angiography. 4.5x4.5-mm peripapillary OCT angiography (OCTA) images of the radial peripapillary capillary (RPC) plexus of the left eye of an individual with frontotemporal dementia (FTD) with average peripapillary capillary flux index (CFI) 0.441 (A) and the left eye of a control with normal cognition with average CFI 0.467 (B). *Zeiss Cirrus HD-5000 Spectral-Domain OCT with AngioPlex OCTA software*, *version 11*.*0*.*0*.*29946 (Carl Zeiss Meditec)*.

Results of GEE analysis of the association between OCTA parameters and FTD diagnosis are shown in Tables 3–5. Both 3x3-mm circle and ring PD (p = 0.02) and circle and ring VD (p = 0.02 and p = 0.01, respectively) were significantly lower in individuals with FTD (Table 3, Fig 2). FAZ area and 6x6-mm PD and VD did not significantly differ between FTD and controls, but there was a trend toward reduced 6x6-mm macular PD and VD in individuals with FTD vs controls (Tables 3 and 4). In FTD, peripapillary CFI was significantly reduced compared to controls (p = 0.01). There was no significant difference in CPD between FTD and controls (p = 0.22) (Table 5, Fig 3).

**Table 3:** Results of OCTA parameters for 3x3-mm circle and ring regions in FTD participants and controls with generalized estimating equation analysis, adjusted for age and sex as covariates.

OCTA Parameter Mean ± SD (N)	FTD	Control	P-value*
**FAZ area**	0.198 ± 0.084 (N = 22)	0.220 ± 0.095 (N = 85)	0.337
**3x3-mm circle PD**	0.329 ± 0.054 (N = 26)	0.355 ± 0.029 (N = 85)	**0.019**
**3x3-mm ring PD**	0.351 ± 0.047 (N = 26)	0.375 ± 0.030 (N = 85)	**0.016**
**3x3-mm circle VD**	18.25 ± 2.92 (N = 26)	19.69 ± 1.64 (N = 85)	**0.017**
**3x3-mm ring VD**	19.24 ± 2.87 (N = 26)	20.76 ± 1.72 (N = 85)	**0.012**

Abbreviations: FAZ, foveal avascular zone; PD, perfusion density; VD, vessel density

*P-value based on test of difference between means using generalized estimating equations (GEE) to account for correlation between eyes of the same patient, adjusted for age and sex.

**Table 4:** Results of OCTA parameters for 6x6-mm circle, inner ring, and outer ring regions in FTD participants and controls with generalized estimating equation analysis, adjusted for age and sex as covariates.

OCTA Parameter Mean ± SD (N)	FTD	Control	P-value*
**6x6-mm circle PD**	0.403 ± 0.061 (N = 24)	0.423 ± 0.041 (N = 85)	0.098
**6x6-mm inner ring PD**	0.398 ± 0.060 (N = 24)	0.417 ± 0.045 (N = 85)	0.111
**6x6-mm outer ring PD**	0.407 ± 0.073 (N = 24)	0.433 ± 0.041 (N = 85)	0.093
**6x6-mm circle VD**	16.59 ± 2.30 (N = 24)	17.14 ± 1.99 (N = 85)	0.172
**6x6-mm inner ring VD**	16.63 ± 2.39 (N = 24)	17.42 ± 1.72 (N = 85)	0.103
**6x6-mm outer ring VD**	16.71 ± 2.71 (N = 24)	17.57 ± 1.49 (N = 85)	0.121

Abbreviations: PD, perfusion density; VD, vessel density

*P-value based on test of difference between means using generalized estimating equations (GEE) to account for correlation between eyes of the same patient, adjusted for age and sex.

**Table 5:** Results of peripapillary OCTA parameter analysis in FTD participants and controls with generalized estimating equation analysis, adjusted for age and sex and covariates.

OCTA Parameter Mean ± SD (N)	FTD	Control	P-value*
**4.5x4.5-mm CPD**	0.430 ± 0.023 (N = 20)	0.434 ± 0.015 (N = 85)	0.216
**4.5x4.5-mm CFI**	0.420 ± 0.038 (N = 20)	0.439 ± 0.031 (N = 85)	**0.014**

Abbreviations: CPD, capillary perfusion density; CFI, capillary flux index

*P-value based on test of difference between means using generalized estimating equations (GEE) to account for correlation between eyes of the same patient, adjusted for age and sex.

## Discussion

The SCP consists of both large and small vessels that supply all other retinal vascular plexuses, including the RPC plexus, which is confined to the peripapillary region [15]. The retinal microvasculature has not been previously described in individuals with FTD; however, several studies have documented quantifiable regional patterns of decreased cerebral blood flow on neuroimaging in AD, FTD, and dementia with Lewy bodies [16, 17]. Shimizu et al. found that cerebral perfusion can vary regionally despite widespread brain atrophy in FTD, with predominant hypoperfusion in the right prefrontal cortex and bilateral medial frontal lobe.

In this cross-sectional study, we found that individuals with FTD had significantly reduced macular 3x3-mm PD and VD as well as peripapillary CFI compared to controls. We also found a trend toward reduced 6x6-mm circle, inner ring, and outer ring PD and VD in individuals with FTD compared to controls. Previous findings have suggested that CFI is a sensitive measure of physiological retinal blood flow changes [18]. The higher lateral resolution of the 3x3-mm scan with improved ability to resolve the retinal microvasculature may contribute to the finding of significance in the 3x3-mm OCTA scans [19, 20]. While the exact mechanism underlying reduced retinal perfusion in patients with FTD is unknown, regional patterns of hypoperfusion similar to those seen in the brain may play a role. Our findings suggest that FTD-mediated neurodegenerative changes may result in a global pattern of retinal hypoperfusion and highlight the value of analyzing both the macular and peripapillary regions in order to characterize microvascular retinal changes in different neurodegenerative diseases [21].

We did not observe a reduction in CPD in FTD compared to controls. Prior studies investigating CPD in AD found increased CPD compared to controls, which may be attributed to amyloid-induced local inflammation and angiogenesis driving the vasodilation of existing capillaries and production of additional retinal capillaries [22]. The absence of amyloid accumulation in FTD and the different pathophysiology of various FTD subtypes may explain the lack of a significant difference in CPD observed in FTD compared to controls [23–25]. Prior studies investigating changes in retinal vascular autoregulatory responses in healthy subjects reported a greater magnitude of change in flux measures compared with density measures, suggesting that CFI may be a more sensitive measure of retinal blood flow than CPD [18].

We did not observe any significant differences in retinal structural measures such as GC-IPL thickness, RNFL thickness, or CST in FTD compared to controls. Reduced RNFL and GC-IPL thickness has been reported in 17 FTD eyes [26], but no significant difference was shown in larger studies that utilized CSF total tau: *β*-amyloid biomarker to exclude AD and categorize FTD patients into subgroups based on molecular pathology (tauopathy, TDP-43, unknown) [27, 28]. GC-IPL thinning has been associated with reduced gray matter volumes in the visual cortex and cerebellum [29] and GC-IPL thinning has been documented in amyotrophic lateral sclerosis, another TDP-43 proteinopathy associated with FTD [28, 30]. As the larger cohort consisted primarily of patients categorized into the tauopathy FTD subgroup, it is possible that both our cohort and the 17-eye cohort represented more patients with TDP-43 proteinopathy, which may explain the observed differences in results. Given that the SCP resides in the ganglion cell layer and supplies all other vascular plexuses, it is possible that the degree of retinal perfusion is linked to GC-IPL thickness such that more significant thinning may be observed later in the disease process as FTD progresses [15, 31]. Future longitudinal studies with larger cohorts of FTD patients categorized into subgroups may further characterize any observed changes in retinal thickness and determine whether these measures are useful biomarkers for differentiating FTD from other neurodegenerative disorders and from controls.

Our study found no significant differences in SFCT or CVI in FTD compared to controls, although, on average, SFCT was thicker in patients with FTD. Choroidal structural changes have not previously been investigated in FTD; however, prior research in healthy eyes has demonstrated that SFCT may vary regionally and with physiologic factors (age, axial length, intraocular pressure, choroidal vascular area, and systolic blood pressure, among others) [32]. Given the greater robustness of CVI as a marker of choroidal vascularity, our results suggest that changes in choroidal structure and vascularity may not be a predominant finding in FTD. Additional work may determine whether measures of choroidal thickness and vascularity have value as biomarkers of FTD.

### Strengths and limitations

This study is the largest imaged cohort of FTD individuals to date that uses both OCT and OCTA to characterize retinal and choroidal microvascular and structural findings. We had a large control group, with an approximately 3:1 control-to-case ratio, excluded potential confounding factors prior to analysis, used high quality images, and used multivariable GEE to account for inclusion of 2 eyes from the same participant and adjusted for age and sex during statistical analysis.

Our study has several inherent limitations. Diagnosis of FTD was determined by experts based on clinical diagnosis and neuroimaging without the use of serum or CSF analysis or genetic biomarkers. This is the current standard of care in neurology due to limitations of existing biomarkers [4]. Diagnosis of FTD may be confounded by its clinical similarity to other neurodegenerative disorders, particularly AD [2, 3]. Autopsy-proven studies have reported pathologically proven AD in 3–17% of clinically diagnosed FTD patients [6]. Future studies evaluating retinal and choroidal findings in FTD based on a combination of diagnostic clinical criteria, serum or CSF analyses, and genetic testing may confirm exclusion of AD and may identify any retinal and choroidal imaging differences between the clinical and pathological subtypes of FTD [6, 28]. Some patients with advanced FTD cannot adequately participate in image acquisition due to difficulty following instructions, fatigue, and fixation errors, as encountered in patients with advanced AD [33, 34]. There was variability in the number of images analyzed for the various retinal parameters measured, with more OCTA images excluded due to the greater susceptibility of OCTA images to motion and other artifact compared to OCT [35]. Additionally, our study did not analyze the deep capillary plexus, as these images are limited by projection artifact from overlying vessels in the SCP and result in a low yield of high-quality images for analysis [36]. Axial length was not measured in study participants; however, we excluded individuals with SEQ < -6D or > +6D in magnitude to minimize any potential impact of image magnification due to axial length variation on OCTA measurements [37]. In addition, given the link between systemic hypertension and retinal microvascular rarefaction, we excluded patients with uncontrolled systemic hypertension [38]; however, we did not account for other systemic vascular diseases such as peripheral vascular disease [39]. Our study was cross-sectional, so we cannot comment on causal relationships between retinal and choroidal findings in FTD or assess progression of disease over time.

In conclusion, we found that individuals with FTD demonstrated significantly reduced peripapillary CFI and 3x3-mm macular VD and PD compared with controls, with a trend toward reduced 6x6-mm macular PD and VD in subjects with FTD. This study is the first to suggest that changes in the retinal microvasculature in FTD patients may be potentially useful as a noninvasive biomarker for diagnosis. This is especially important for patients with FTD given the heterogenous clinical presentations, biomarker limitations, and necessity for histological examination for definite diagnosis. Future studies to compare FTD patients to individuals with other neurodegenerative disorders, compare the different clinical subgroups of FTD, and investigate longitudinal changes in FTD are necessary to further evaluate the clinical significance of OCT and OCTA in improving diagnosis and hopefully ultimately quality of life for patients with FTD, as well as their caregivers.

## Supporting information

S1 Data Supplementary data sheet. Deidentified FTD and control participant data sheet.
(XLSX)
