# Correction: Angioarchitectural alterations in the retina and choroid in frontotemporal dementia

**DOI:** 10.1371/journal.pone.0314692

**Published:** 2024-11-26

**Authors:** Ariana Allen, Cason B. Robbins, Suzanna Joseph, Angela Hemesath, Anita Kundu, Justin P. Ma, Alice Haystead, Lauren Winslow, Rupesh Agrawal, Kim G. Johnson, Andrea C. Bozoki, Sandra S. Stinnett, Dilraj S. Grewal, Sharon Fekrat

The fourth author’s name is spelled incorrectly. The correct name is: Angela Hemesath. The correct citation is: Allen A, Robbins CB, Joseph S, Hemesath A, Kundu A, Ma JP, et al. (2024) Angioarchitectural alterations in the retina and choroid in frontotemporal dementia. PLOS ONE 19(11): e0312118. https://doi.org/10.1371/journal.pone.0312118.

## References

[1] Allen A, Robbins CB, Joseph S, Hemesat A, Kundu A, Ma JP, et al. (2024) Angioarchitectural alterations in the retina and choroid in frontotemporal dementia. PLOS ONE 19(11): e0312118. 10.1371/journal.pone.0312118 39514529 PMC11548753

